# The Pressor Response to Concurrent Stimulation of the Mesencephalic Locomotor Region and Peripheral Sensory Afferents Is Attenuated in Normotensive but Not Hypertensive Rats

**DOI:** 10.3389/fphys.2019.00095

**Published:** 2019-02-13

**Authors:** Nan Liang, Gary A. Iwamoto, Ryan M. Downey, Jere H. Mitchell, Scott A. Smith, Masaki Mizuno

**Affiliations:** ^1^Department of Health Care Sciences, University of Texas Southwestern Medical Center, Dallas, TX, United States; ^2^Department of Human Health Sciences, Graduate School of Medicine, Kyoto University, Kyoto, Japan; ^3^Department of Cell Biology, University of Texas Southwestern Medical Center, Dallas, TX, United States; ^4^Department of Internal Medicine, University of Texas Southwestern Medical Center, Dallas, TX, United States; ^5^Department of Pharmacology and Physiology, Georgetown University, Washington, DC, United States

**Keywords:** hypertension, mesencephalic locomotor region, central command, exercise pressor reflex, blood pressure, sympathetic nerve activity

## Abstract

Central command (CC) and the exercise pressor reflex (EPR) regulate blood pressure during exercise. We previously demonstrated that experimental stimulation of the CC and EPR pathways independently contribute to the exaggerated pressor response to exercise in hypertension. It is known that CC and EPR modify one another functionally. Whether their interactive relationship is altered in hypertension, contributing to the generation of this potentiated blood pressure response, remains unknown. To address this issue, the pressor response to activation of the CC pathway with and without concurrent stimulation of the EPR pathway, and vice versa, was examined in normotensive Wistar Kyoto (WKY) and spontaneously hypertensive (SHR) rats. In decerebrated, paralyzed animals, activation of the CC pathway was evoked by electrical stimulation of the mesencephalic locomotor region (MLR; 20–50 μA in 10-μA steps). Electrical stimulation of the sciatic nerve (SN, 3, 5, and 10 × motor threshold; MT) was used to activate hindlimb afferents known to carry EPR sensory information. In both WKY and SHR, the algebraic sum of the pressor responses to individual stimulation of the MLR and SN were greater than when both inputs were stimulated simultaneously. Although the blood pressure response to a constant level of SN stimulation was not significantly affected by concurrent MLR stimulation at variable intensities, the pressor response to a constant level of MLR simulation was significantly attenuated by concurrent SN stimulation in WKY but not in SHR. These findings suggest the interactive relationship between CC and the EPR is inhibitory in nature in both WKY and SHR. However, the neural occlusion between these central and peripheral pressor mechanisms is attenuated in hypertension.

## Introduction

Neural drives from higher brain centers (central command, CC) and peripheral skeletal muscles (the exercise pressor reflex, EPR) contribute to the regulation of arterial blood pressure (ABP) during exercise. CC activates cardiovascular as well as locomotor control circuits simultaneously (Eldridge et al., 1985; Gandevia and Hobbs, 1990; Bedford et al., 1992; Gandevia et al., 1993; Tsuchimochi et al., 2009; Matsukawa et al., 2011; Nakamoto et al., 2011; Liang et al., 2016), playing a crucial role in mediating the circulatory responses to physical activity (Goodwin et al., 1972; Matsukawa, 2012; Michelini et al., 2015). The mechanosensitive and metabosensitive components of the EPR, which are activated by stimulating group III/IV skeletal muscle afferent fibers (Kaufman et al., 1984), also reflexively increase ABP and heart rate (HR) during exercise while the larger diameter group I/II fibers do not (McCloskey and Mitchell, 1972). Evidence suggests these central and peripheral neural signals converge both in the spinal cord as well as within cardiovascular centers in the brainstem to regulate the circulatory system in an integrative fashion (Waldrop et al., 1986; Rybicki et al., 1989; Degtyarenko and Kaufman, 2000a,b,c, 2005).

Hypertension is one of the most important causes of premature morbidity and mortality contributing to a number of cardiovascular related disorders (Kearney et al., 2005). Exercise is known to improve cardiovascular health and reduce resting blood pressure (Gibbons et al., 2002; Pescatello et al., 2004). However, circulatory hemodynamics are abnormally potentiated during exercise in this disease limiting the intensity and duration of physical activity that can be safely prescribed (Smith, 2010). The mechanisms underlying this overactive cardiovascular responsiveness are not fully understood. Recent studies in our laboratory suggest that dysfunction in both CC and the EPR contribute significantly to the exaggerated cardiovascular response to exercise in hypertension. Specifically, electrical stimulation of the mesencephalic locomotor region (MLR), a putative component of the CC pathway, induces larger ABP and renal sympathetic nerve activity (RSNA) responses in hypertensive rats compared to normotensive animals (Liang et al., 2016). Likewise, selective activation of the EPR elicits markedly greater increases in mean arterial pressure (MAP) and RSNA in hypertensive compared to normotensive rats (Smith et al., 2006; Leal et al., 2008; Mizuno et al., 2011a,b; Murphy et al., 2013). As stated, it is known that CC and the EPR interact to modulate each other’s activity. Therefore, in addition to each input independently driving the abnormally enhanced cardiovascular response to exercise in hypertension, it is also plausible that alterations in the integrative relationship between the two contribute to this hyper-responsiveness.

The independent contributions of CC and the EPR to cardiovascular regulation during exercise has been studied extensively. In contrast, investigations designed to elucidate the interactive behavior of these inputs have been studied far less. The few studies that have investigated this interaction suggest that the relationship between CC and the EPR is inhibitory in nature (i.e., the sum of the pressor responses to independent activation of each is greater than the response elicited when both are stimulated simultaneously) (Degtyarenko and Kaufman, 2000a,b,c). To date, however, no studies have been conducted to determine whether this interactive relationship is altered with the pathogenesis of hypertension.

The purpose of this study, therefore, was to determine the interactive relationship between CC and the EPR in both normal, healthy rats and hypertensive animals. We hypothesized that the previously established inhibitory interaction between the two neural inputs, important for blood pressure regulation, is reduced in hypertensive animals as compared to normotensive controls. To test this hypothesis, we examined the integrative ABP response to peripheral and central neural activation in two distinct ways: (1) during electrical stimulation of peripheral skeletal muscle afferent fibers (sensory neurons known to be part of the EPR pathway) at multiple intensities throughout activation of the MLR (a component of the CC pathway) at a single, constant intensity, and (2) during MLR stimulation at multiple intensities throughout activation of skeletal muscle afferent fibers at a single, constant intensity. Both paradigms were performed in decerebrate, paralyzed normotensive Wistar-Kyoto (WKY) and spontaneously hypertensive (SHR) rats. These paradigms were chosen as stimulation of peripheral afferent fibers and the MLR are common strategies used to investigate the contributions of the EPR and CC, respectively, to cardiovascular regulation in rodents (Bedford et al., 1992; Koba et al., 2005, 2006a,b, 2014; Liang et al., 2016).

## Materials and Methods

Experiments were performed using age-matched (13–16 weeks) male WKY (*n* = 11) and SHR (*n* = 11) rats. Animals were maintained in a temperature-controlled environment, fed ad libitum, and kept on a 12-h light-dark cycle. All studies were performed in accordance with the United States Department of Health and Human Services NIH *Guide for the Care and Use of Laboratory Animals.* The procedures outlined were approved by the Institutional Animal Care and Use Committee of the University of Texas Southwestern Medical Center.

### General Surgical Preparation

As described previously (Smith et al., 2001; Liang et al., 2016), animals were anesthetized with isoflurane gas (4% in 100% oxygen, 1.5–2% during surgery) and intubated for mechanical ventilation. Fluid-filled polyurethane catheters were inserted into both common carotid arteries for the measurement of ABP and MAP (MLT0380/D; ADInstruments) and into the right external jugular vein for the administration of drugs. A continuous infusion of 1 M NaHCO3, 5% dextrose Ringer solution was established via the jugular vein at a rate of 3–5 ml h^-1^ kg^-1^ to stabilize fluid balance and maintain baseline ABP (Quintin, 1990). Electrocardiograph signals (ECG) were recorded by placing needle electrodes on the back of the animal, and HR was derived from the R wave of the ECG recording. ABP and HR were continuously monitored. Respiratory thoracic movement was visually observed and rectal temperature was maintained between 36.5 and 38.0 degrees Celsius with a heating pad and an external lamp throughout the experiment. All animals were held in a stereotaxic head unit (Kopf Instruments), and a pre-collicular decerebration was performed rendering the animals insentient. Dexamethasone (0.2 mg) was given intravenously to minimize brain edema. Gas anesthesia was discontinued immediately following the decerebration procedure. Experimental protocols were performed at least 1.25 h thereafter (Kohn, 1997).

### MLR Stimulation (to Mimic CC Activation)

The experimental procedures used for MLR stimulation have been described previously (Liang et al., 2016). Briefly, a concentric bipolar electrode (outer pole diameter: 200 μm, stainless steel; inner pole wire diameter: 50 μm, platinum/iridium; FHC Inc.) connected to a photoelectric stimulus isolation unit and stimulator (Grass S88, Grass Instrument Co.) was used. The tip of the electrode was placed 1.7–2.0 mm lateral, 0.3–0.8 mm anterior, and 3.5–4.5 mm deep from the surface junction of the superior and inferior colliculi (Bedford et al., 1992; Koba et al., 2005, 2006a,b, 2014; Liang et al., 2016). The motor threshold (MT) of MLR stimulation was determined by slightly increasing the current intensity until movement of the animal was observed. The site of the MLR stimulation was identified by physiological criteria as previously reported (Bedford et al., 1992; Koba et al., 2005, 2006a,b, 2014; Liang et al., 2016).

### Sciatic Nerve Stimulation (to Mimic EPR Activation)

Electrical stimulation of the sciatic nerve (SN) was utilized to activate skeletal muscle afferent fibers. The left SN was exposed and separated from surrounding tissue at the knee joint. The nerve bundle was mounted on a bipolar electrode of Ag-AgCl wires, which connected to a photoelectric stimulus isolation unit and stimulator (Grass S88, Grass Instrument Co.), in a warm mineral oil pool surrounded with connective tissue and skin. The MT of SN stimulation was determined by slightly increasing the current intensity until muscle contraction was induced and movement was observed in the left hindlimb. The rat was paralyzed with pancuronium bromide (1 mg kg^-1^, i.v.), and the lungs were artificially ventilated with a respirator after MT determination for SN and MLR stimulation.

### Recording of Tibial Nerve Discharge

Tibial nerve discharge (TND) was recorded to assess motor activity induced by electrical stimulation of the MLR in one decerebrate, paralyzed WKY and SHR rat. As previously reported (Liang et al., 2016), the left tibial nerve was separated from the SN at the knee joint. To eliminate afferent discharge, the distal portion of the tibial nerve was ligated. The nerve bundle was mounted on a bipolar electrode of Ag-AgCl wires in a warm mineral oil pool surrounded with connective tissue and skin. The original TND was amplified with a band-pass filter at 100–4,000 Hz, then full-wave rectified.

### Recording of Dorsal Root Nerve Activity

To identify which groups of afferent nerve fibers were activated by SN stimulation, compound action potentials of dorsal root neural activity (DRNA) were recorded in one decerebrate, paralyzed WKY and SHR rat to assess sensory nerve activity induced by electrical stimulation of the SN nerve. A laminectomy exposing the lower limb portions of the spinal cord (L_2_–L_6_) was performed as previously described (Smith et al., 2001, 2006; Mizuno et al., 2011a,b, 2015). The dura layers surrounding the cord were cut and reflected. The L_4_ and L_5_ dorsal roots were carefully isolated and sectioned. The cut peripheral ends of the roots were placed on bipolar platinum electrodes. The exposed neural tissue was immersed in mineral oil. The original compound action potentials of DRNA was amplified with a band-pass filter at 100–4,000 Hz. The distance between the stimulating and recording electrodes was assessed along with the latency of the responses in order to calculate conduction velocity. Conduction velocity at 31–120 m/s was classified as group I/II fibers, those from 2.6 to 30 m/s as group III, and those with less than 2.5 m/s as group IV (Mitchell, 1985).

### Experimental Protocols

In protocols in which MLR or SN stimulation were applied alone, the following parameters were utilized. In MLR stimulation, current intensities of 20, 30, 40, and 50 μA (pulse duration of 1 ms at 60 Hz, for 30 s) were used in accordance with earlier studies (Bedford et al., 1992; Degtyarenko and Kaufman, 2005; Koba et al., 2005, 2006a,b, 2014; Liang et al., 2016). Regarding SN stimulation, current intensities equal to 3, 5, and 10 times MT (pulse duration of 0.75 ms at 20 Hz, for 30 s) were used. The latter intensities have been shown to be sufficient for activation of Group I-IV afferent fibers (McCallister et al., 1986; Harms et al., 2016).

In combined activation protocols, the MLR and SN were stimulated concurrently. In one paradigm, the current intensity used for SN stimulation was fixed at 3 × MT while MLR stimulation was applied over a range of 20–50 μA. In a second paradigm, the current intensity of MLR stimulation was fixed at 40 μA while SN stimulation was applied over a range of 3–10 × MT. When administered over a range, the application of current intensity was randomized. Moreover, the combined stimulation protocols were always performed after the sole stimulation protocols. The inter-protocol interval was at least 5 min between stimulations. All protocols were performed in paralyzed, decerebrate rats. If voluntary ventilation and/or movement were observed, supplemental doses of pancuronium bromide (0.5–0.75 mg kg^-1^, i.v.) were administered.

At the conclusion of all experiments, the insentient animals were humanely killed by intravenous injection of saturated potassium chloride (4 M, 2 ml/kg iv). The heart and lungs were excised and weighed. In addition, the tibia was harvested and the length measured.

### Data Acquisition and Analysis

ABP, MAP, HR, and stimulation pulse data were recorded and analyzed using data acquisition software (LabChart, ADInstruments) for the Powerlab analog-to-digital convertor (Powerlab8/30; ADInstruments) at a 1-kHz sampling rate. The TND and DRNA were recorded at a sampling rate of 4-kHz. Data sets of 1 s averages for MAP and HR were analyzed. Baseline values were determined by evaluating 30 s of recorded data immediately before the MLR and/or SN stimulation. The maximum response of each variable was defined as the peak change from baseline elicited by electrical stimulation.

### Statistical Analyses

Data were analyzed using Student’s unpaired *t*-tests (WKY vs. SHR), two-way repeated measures ANOVA (rat group and MLR or SN stimulation intensity) with rat group (WKY and SHR) as a within-subject factor. If significant interaction and main effects were obtained with ANOVA, *post hoc* analyses were performed using a Student’s unpaired *t*-test with Holm’s sequential Bonferroni correction applied (Holm, 1979). The level of statistical significance was defined as *P* < 0.05. Results are presented as means ± *SE*.

## Results

Morphometric characteristics, baseline hemodynamics and MLR and SN motor thresholds for WKY and SHR are summarized in Table 1. There were no significant differences in body weight or lung weight-to-body weight ratios between groups. As previously reported (Smith et al., 2006; Leal et al., 2008; Mizuno et al., 2011a,b, 2014; Murphy et al., 2013; Liang et al., 2016), heart weight-to-body weight ratios as well as heart weight-to-tibial length ratios were significantly greater in SHR than WKY. Consistent with our previous study (Liang et al., 2016), baseline HR was significantly lower and baseline MAP was significantly higher in SHR compared to WKY. There were no statistical differences in MT for either MLR or SN stimulation between groups.

**Table 1:** Morphometric characteristics, baseline hemodynamics and motor threshold.

	WKY	SHR
*N*	11	11
Body weight, g	328 ± 3	327 ± 4
MAP, mmHg	72 ± 4	107 ± 7*
HR, beats min^-1^	465 ± 11	415 ± 11*
Heart weight/body weight, mg/g	3.0 ± 0.1	3.3 ± 0.1*
Heart weight/tibial length, mg/mm	25.0 ± 1.2	27.9 ± 0.4*
Lung weight/body weight, mg/g	5.3 ± 0.3	5.9 ± 0.3
MLR stimulation motor threshold, μA	21 ± 2	23 ± 2
SN stimulation motor threshold, μA	49 ± 5	50 ± 4


*Values are means ± SE WKY, Wistar-Kyoto rats; SHR, spontaneously hypertensive rats; MAP, mean arterial pressure; HR, heart rate; MLR, mesencephalic locomotor region; SN, sciatic nerve. ^∗^*P* < 0.05 compared with WKY rats.*

**FIGURE 1:**
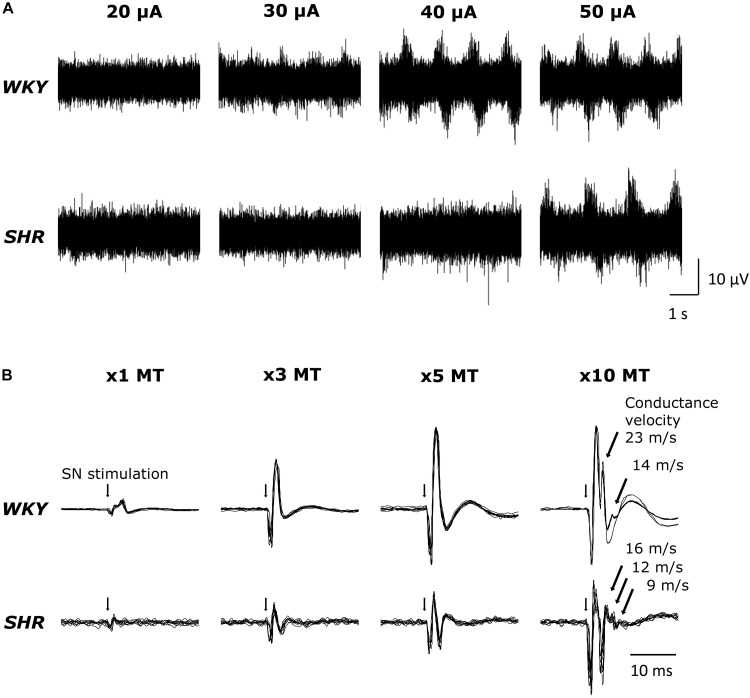
**(A)** Original tracings demonstrating tibial nerve discharge (TND) in response to mesencephalic locomotor region (MLR) stimulation (20–50 μA) in WKY (MT: 18.3 μA) and SHR (MT: 18.9 μA). **(B)** Original tracings (superimposed 10 trials for each condition) of dorsal root nerve activity (DRNA) in response to stimulation of the sciatic nerve (SN) (1–10 × MT) in WKY and SHR.

Original tracings of TND in response to MLR stimulation from one representative of both groups of animals are shown in Figure 1A. MLR stimulation increased TND in an intensity dependent manner as previously reported (Liang et al., 2016). Superimposed DRNA recordings (10 traces) in response to SN stimulation from one WKY and one SHR are shown in Figure 1B. SN stimulation significantly increased DRNA in an intensity-dependent manner in both animals, while it seemed the magnitude of the responses were somewhat smaller in SHR than WKY at all current intensities. Responses with fast conduction velocity, attributable to activation of group I/II afferent fibers, were clearly detected at current intensities of 1,3 and 5 × MT. At the higher intensity of 10 × MT, the response with a slow conduction velocity, attributable to group III/IV afferent fibers, could also be observed (conduction velocity ranged 14–23 m/s in WKY and 9–16 m/s in SHR).

**FIGURE 2:**
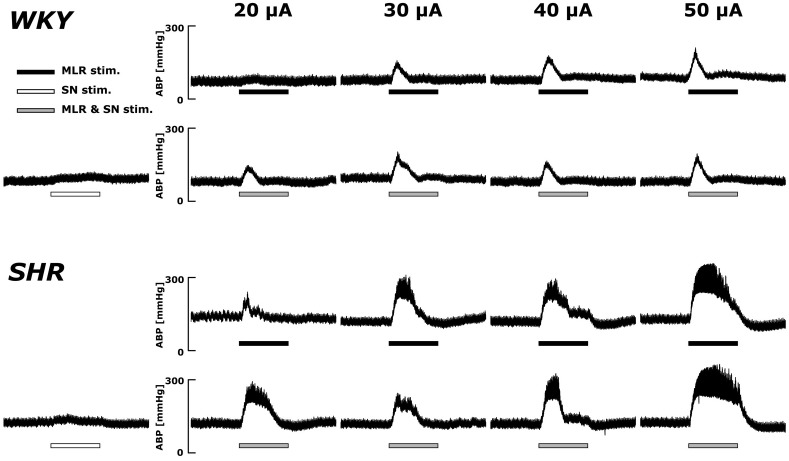
Representative recordings of the arterial blood pressure (ABP) response to MLR stimulation with or without concurrent SN stimulation (3 × MT) in WKY (MLR stimulation MT: 37.5 μA) and SHR (MLR stimulation MT: 24.1 μA). Horizontal bars indicate the 30-s period of each stimulation; black: MLR stimulation alone; white: SN stimulation alone; gray: MLR and SN stimulation.

Original ABP tracings in response to MLR stimulation with and without SN stimulation in representative WKY and SHR are presented in Figure 2. As previously reported (Liang et al., 2016), the ABP responses to MLR stimulation alone were markedly greater in SHR compared to WKY across all stimulation intensities. The responses to MLR stimulation were not appreciably affected by concomitant SN stimulation in either WKY or SHR.

**FIGURE 3:**
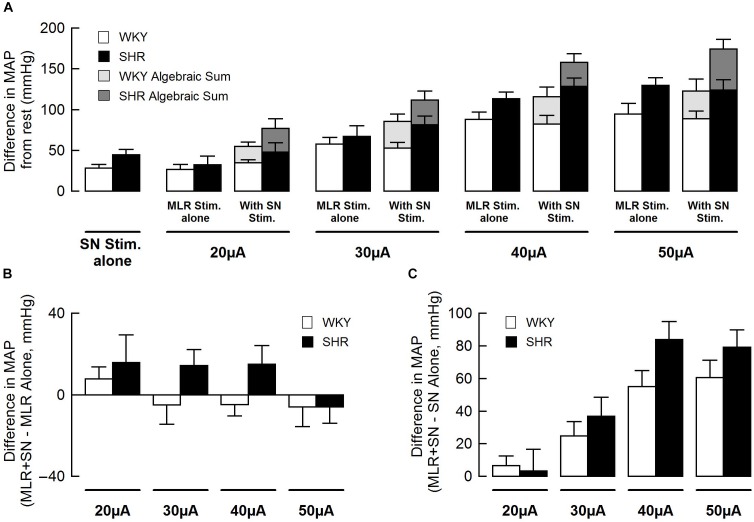
**(A)** Peak changes in mean arterial pressure (MAP) associated with MLR and/or SN stimulation in WKY (white bars, *n* = 11) and SHR (black bars, *n* = 11). The intensity of the SN stimulation was fixed at 3 × MT with the MLR stimulation ranging from 20 to 50 μA. Light (WKY) and dark (SHR) gray bars depict the algebraic sum of MAP responses to SN stimulation alone + MLR stimulation alone. **(B)** Differences in the MAP response between combined stimulation and MLR stimulation alone. **(C)** Differences in the MAP response between combined stimulation and SN stimulation alone.

Figure 3 summarizes group mean responses to MLR stimulation with or without concurrent SN stimulation in WKY and SHR. In hypertensive animals, MAP responses to stimulation of the MLR were greater compared to normotensive rats (Figure 3A; main “rat group” effect, *P* = 0.08 for MLR stimulation alone; main “rat group” effect*, P* < 0.01 for MLR + SN stimulation). In addition, MAP responses to MLR stimulation increased with stimulus intensity in both SHR and WKY (Figure 3A; main “stimulation intensity” effect, *P* < 0.01 for MLR stimulation alone; main “stimulation intensity” effect, *P* < 0.01 for MLR +SN stimulation). Importantly, in both groups of animals, the algebraic sum of the MAP response (i.e., SN stimulation alone + MLR stimulation alone) was larger than the MAP response evoked during simultaneous activation of the SN and MLR at all intensities tested indicative of an inhibitory interaction between the two inputs (Figure 3A). The difference in MAP calculated as the MAP evoked during MLR+SN stimulation minus the MAP evoked during MLR stimulation alone was not significantly different between WKY or SHR at any stimulus intensity (Figure 3B). Likewise, the difference in MAP calculated as the MAP evoked during MLR+SN stimulation minus the MAP evoked during SN stimulation alone was not significantly different between WKY or SHR at any stimulus intensity (Figure 3C).

**FIGURE 4:**
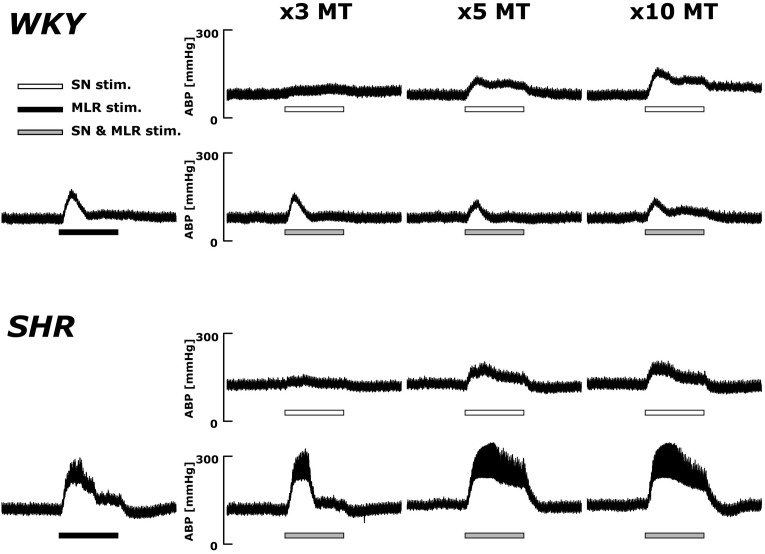
Representative recordings of the blood pressure response to SN stimulation with or without concurrent MLR stimulation (40 μA) in WKY and SHR (identical rats as shown in the Figure 2). Horizontal bars indicate the 30-s period of each stimulation; white: SN stimulation alone; black: MLR stimulation alone; gray: MLR and SN stimulation.

Original ABP tracings in response to SN stimulation with and without MLR stimulation in representative WKY and SHR are presented in Figure 4. The pressor responses to SN stimulation increased in an intensity-dependent manner in both animal groups. Importantly, as compared with SN stimulation alone, the pressor responses became smaller with increased SN stimulation intensity when combined with MLR stimulation in WKY but became surprisingly larger in SHR.

**FIGURE 5:**
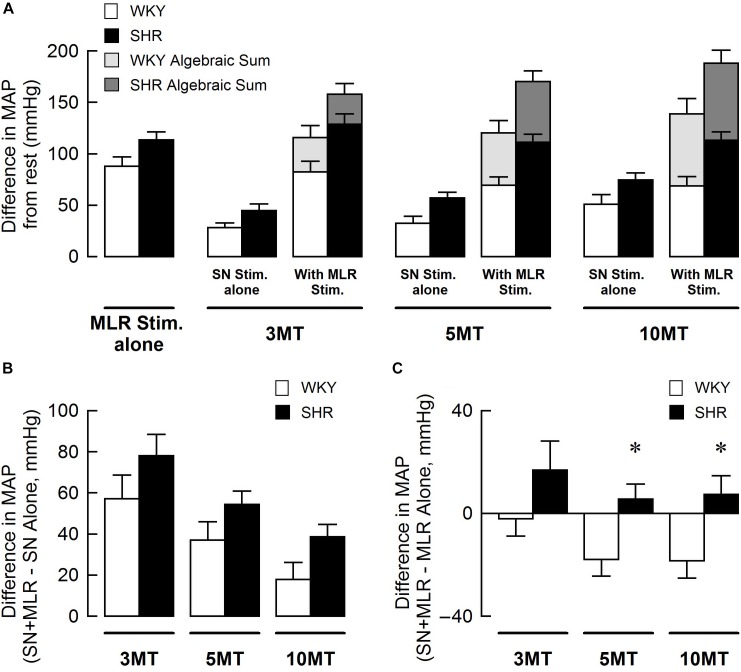
**(A)** Peak changes in mean arterial pressure (MAP) in response to MLR and/or SN stimulation in WKY and SHR. The intensity of the MLR stimulation was fixed at 40 μA with SN stimulation ranging from 3 to 10 × MT. Light (WKY, *n* = 11) and dark (SHR, *n* = 11) gray bars depict the algebraic sum of MAP responses to SN stimulation alone + MLR stimulation alone. **(B)** Differences in the MAP response between combined stimulation and SN stimulation alone. **(C)** Differences in the MAP response between combined stimulation and MLR stimulation alone. ^∗^*P* < 0.05 significant difference between WKY and SHR.

Figure 5 summarizes group mean responses to SN stimulation with or without simultaneous MLR stimulation in WKY and SHR. In SHR, MAP responses to stimulation of the SN were significantly greater compared to WKY (Figure 5A; main “rat group” effect*, P* < 0.05 for SN stimulation alone; main “rat group” effect*, P* < 0.01 for SN + MLR stimulation). In addition, the pressor responses to SN stimulation increased with each elevation in stimulus intensity in both SHR and WKY (Figure 5A; main “stimulation intensity” effect, *P* < 0.01 for SN stimulation alone) while decreasing step-wise when combined with MLR stimulation (Figure 5A; main “stimulation intensity” effect, *P* < 0.01 for SN+MLR stimulation). As before, in each group of animals, the algebraic sum of the MAP response (i.e., SN stimulation alone + MLR stimulation alone) was larger than the pressor response evoked during concurrent activation of the SN and MLR at all intensities tested. Evidence again that an inhibitory interaction exists between the two inputs (Figure 5A). The difference in MAP calculated as the MAP evoked during SN+MLR stimulation minus the MAP evoked during SN stimulation alone tended to be greater in SHR compared to WKY (Figure 5B; *P* = 0.08). Additionally, the differences in MAP calculated as the MAP evoked during SN+MLR stimulation minus the MAP evoked during MLR stimulation alone were significantly higher in SHR than WKY (Figure 5C, *P <* 0.05) with the differences actually below baseline in WKY. The latter finding suggests that the inhibitory relationship between the two inputs is maintained in normotensive animals but compromised in hypertensive rats.

**FIGURE 6:**
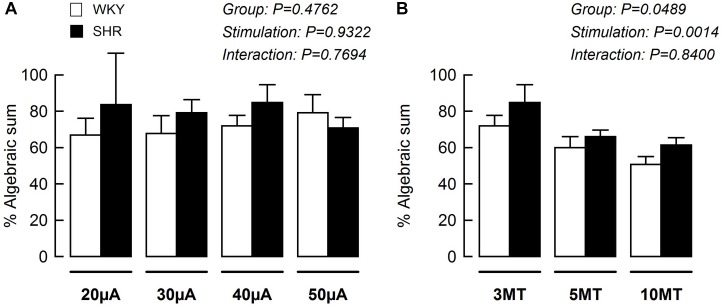
The mean arterial pressure (MAP) response to concurrent MLR and SN stimulation expressed as a percentage of the algebraic sum of the MAP responses to SN stimulation alone + MLR stimulation alone in WKY (white bars, *n* = 11) and SHR (black bars, *n* = 11). Representations calculated from Figure 3A, 5A. **(A)** The intensity of the SN stimulation was fixed at 3 × MT while MLR stimulation ranged from 20 to 50 μA. **(B)** The intensity of the MLR stimulation was fixed at 40 μA while SN stimulation ranged from 3 to 10 × MT.

Using data from Figure 3A, 5A, the blood pressure responses to combined stimulation of the MLR and SN were calculated as a percent of the algebraic sum of each input alone (i.e., SN stimulation only + MLR stimulation only). The MAP responses evoked during SN stimulation (fixed at 3 × MT) combined with MLR stimulation (ranging from 20 to 50 μA) tended to be a larger percentage of the algebraic sum in SHR compared to WKY at most intensities tested although statistical significance was not reached (Figure 6A). The responses elicited during SN stimulation (ranging from 3 to 10 × MT) combined with MLR stimulation (fixed at 40 μA) were a significantly greater percentage of the algebraic sum in hypertensive compared to normotensive animals (Figure 6B) indicative of a change in the interactive relationship in SHR. In both paradigms, the responses produced during combined stimulation were less than 100% of the algebraic sum indicating an inhibitory relationship existed.

## Discussion

The present study was designed to investigate the interactive relationship between CC and the EPR in the regulation of blood pressure in normotensive and hypertensive animals. Activation of the CC pathway was induced by electrical stimulation of the MLR, while activation of skeletal muscle afferent fibers (components of the EPR pathway) was evoked by electrically stimulating the SN. In normotensive, healthy rats, the findings complement previous reports that the EPR and CC integrate in an inhibitory fashion such that the response to their combined stimulation is less than the algebraic sum of their individual responses. Some reports have described this as inhibition whereas others occlusion (Degtyarenko and Kaufman, 2000a,b,c, 2005; Eldridge et al., 1985; Waldrop et al., 1986; Rybicki et al., 1989). Constituting a major new finding, the data suggest that this inhibitory relationship is compromised in hypertensive animals. It has been previously reported that the independent function of the EPR and CC are exaggerated during exercise in hypertension. The findings from this investigation suggest that this overactivity may result, at least in part, from a reduction in the inhibitory relationship between the two inputs. Stated simply, CC overactivity in hypertension may be due, at least in part, to a reduction in the ability of the EPR to buffer its function and vice versa. This loss of inhibition may partially underlie each inputs abnormal contribution to the exaggerated cardiovascular response to exercise in hypertension.

### TND Response to MLR Stimulation

Central command simultaneously activates neural circuits modulating locomotion as well as cardiovascular function. To preferentially assess CC in the absence of input from the EPR in the current study, it was necessary to apply MLR stimulation after the induction of neuromuscular blockade. This allowed activation of circuits in the CC pathway independent of actual muscle contraction which would, if allowed to occur, concurrently stimulate the EPR. The production of “fictive” locomotion in this manner is a common strategy used in animals to assess CC function (Bedford et al., 1992). To ensure that MLR-induced “fictive” locomotion was equivalent between WKY and SHR, the TND response to MLR stimulation was assessed. Consistent with our previous investigation (Liang et al., 2016), MLR stimulation increased TND in an intensity dependent manner in both groups (Figure 1A). Moreover, as previously reported (Liang et al., 2016), there was no difference in the MT during MLR stimulation when comparing WKY and SHR (Table 1). These data suggest that the motor command evoked by stimulation of the MLR was not different between WKY and SHR.

### DRNA Response to SN Stimulation

Sciatic nerve stimulation at 3, 5, and 10 × MT was utilized to activate skeletal muscle afferent fibers associated with the EPR in this study. To confirm that the afferent fibers were activated by the current intensities used, we recorded DRNA in both WKY and SHR during SN stimulation (Figure 1B). Since sensory threshold is considerably lower than MT in general, a small but distinct response was detected in the DRNA recording with the current intensity at 1 × MT (an intensity used solely to establish MT). Although the magnitude of the DRNA responses at each current intensity was somewhat smaller in SHR than in WKY, DRNA increased in an intensity-dependent manner in both groups. Importantly, it was determined that the responses with fast conduction velocity (characteristic of group I/II afferent fibers) were detectable during SN stimulation of 1-5 × MT (50–100 m/s), while those with slow conduction velocity (characteristic of group III/IV afferent fibers) appeared with 10 × MT in both WKY and SHR (9–23 m/s). This result is consistent with recent studies demonstrating that 5 × MT stimulation activates group I/II or III afferent fibers but not group IV (Harms et al., 2016) in rats. In addition, there was no difference in the latency of responses between WKY and SHR. These data suggest that SN stimulation-induced equivalent afferent fiber activation at all levels of intensity in both groups.

### Impact of SN Stimulation on the Pressor Responses to MLR Stimulation

Consistent with our previous report (Liang et al., 2016), intensity-dependent pressor responses to MLR stimulation were greater in SHR compared to WKY with or without concurrent SN stimulation. Although an inhibitory interaction between the two inputs was evident and tended to be greater in magnitude in WKY compared to SHR (Figure 3B, 6A), the differences were not statistically significant. This might be explained by the intensity of SN stimulation used in this particular protocol (i.e., SN stimulation of 3 × MT). Given this trend, use of a greater SN stimulus than 3 × MT may have elicited a larger occlusive response in WKY compared to SHR. Future investigation is warranted.

### Impact of MLR Stimulation on the Pressor Responses to SN Stimulation

The present study also examined the effects of peripheral afferent input of multiple intensities on the pressor response to activation of the CC pathway (at a constant level) in both hypertensive and normotensive rats. In this paradigm, intensity-dependent pressor responses to SN stimulation were again larger in SHR compared to WKY with or without concurrent MLR stimulation. Importantly, the inhibitory interaction between the two inputs was clear (established by virtue of the pressor response to combined activation of each input being less than the algebraic sum of the response to each input stimulated individually) with the magnitude of the inhibition being significantly greater in WKY compared to SHR (Figure 5B, 6B). As more evidence, the pressor response with combined MLR and SN stimulation remained unchanged or was larger than responses with MLR stimulation alone in SHR but was significantly smaller in WKY (especially at higher SN current intensities). Combined, these analyses suggest that the inhibitory relationship between the EPR and CC was significantly compromised in SHR as compared to WKY.

There is a substantial body of information in normal, healthy animals which suggests that the present results are consistent with prior observations. Stimulation of the MLR is known to produce an inhibition of the activity of cells receiving input from afferents mediating the pressor response to muscle contraction (Degtyarenko and Kaufman, 2000a,b,c). It is probable that this inhibition is not based on presynaptic occlusion of group III/IV afferent terminals but rather postsynaptic inhibition of interneurons (Rudomin and Schmidt, 1999). It is likely that MLR stimulation also inhibits responses due to the tonic activity of high threshold afferents resulting in less overall activity. Given the findings of the investigation, it seems probable that these mechanisms for inhibition were operative in normotensive WKY to a greater extent than in hypertensive animals. Alternatively, it is also possible that stimulation of peripheral afferent neurons in the EPR pathway directly inhibit neurons in the CC pathway centrally contributing to the responses obtained. This is purely speculative, however, as the current study was not designed to make this determination. What is clear from this study is that when the two pathways are stimulated concurrently, the interaction between these central and peripheral blood pressure mechanisms is occlusive in nature albeit attenuated in hypertensive animals.

### Methodological Considerations

Autonomic adjustments regulating the cardiovascular system during exercise are determined by integrating input from the arterial baroreflex as well as the EPR and CC. Moreover, the baroreflex is known to modulate the activity of the EPR and CC. In the current study, the baroreflex remained intact and was not experimentally controlled. Moreover, it has been shown that the sensitivity of the baroreflex is reduced in hypertension (Moreira et al., 1992; Lanfranchi and Somers, 2002; Minami et al., 2003). That being acknowledged, previous studies have demonstrated that the cardiovascular responses to SN stimulation are enhanced in SHR compared to WKY and independent of impairments in baroreflex function (Ruggeri et al., 2000). As such, although an intact baroreflex may have influenced the results observed, its impact would be expected to be minimal.

## Conclusion

The blood pressure response to exercise is abnormally exaggerated in hypertension. Due to the dangers inherent with such an enhanced pressor response, the prescription of physical activity as a safe treatment for this disease is often limited to exercise of short duration and mild to moderate intensity. Determining the mechanisms underlying this exaggerated responsiveness may lead to the development of therapies aimed at reducing this limitation allowing the benefits of exercise to be more fully realized in this patient population. To this end, previous studies have demonstrated that, when activated individually, stimulation the CC pathway and activation of the EPR pathway contribute significantly to the potentiated blood pressure response to exercise in this disease. Importantly, findings from the current study suggest that the CC and EPR overactivity manifest in hypertension is not solely due to alterations in the neural pathways of each input but also from alterations in the manner in which the inputs interact. Specifically, this investigation demonstrated for the first time, that the ability of each input to buffer the activity of the other is compromised in hypertension. This type of reduction in inhibitory influence with the pathogenesis of hypertension is likely to mediate, in part, the exaggerated blood pressure response to activation of both CC and the EPR during physical activity.

## Author Contributions

NL, GI, JM, SS, and MM decided on the conception and design of the research, and interpreted the results of experiments. NL, GI, RD, and MM performed the experiments. NL and MM analyzed the data, prepared the figures, and drafted the manuscripts. NL, GI, RD, JM, SS, and MM approved final version of the manuscripts.

## Conflict of Interest Statement

The authors declare that the research was conducted in the absence of any commercial or financial relationships that could be construed as a potential conflict of interest.
